# Ethyl 4-[(2-hy­droxy­eth­yl)amino]-2-(4-meth­oxyphen­yl)-1-methyl-5-oxo-2,5-di­hydro-1*H*-pyrrole-3-carboxyl­ate

**DOI:** 10.1107/S2414314624012227

**Published:** 2024-12-20

**Authors:** Fatin Nur Ain Abdul Rashid, Mohd Fazli Mohammat, Mohd Abdul Fatah Abdul Manan, Ayisy Amirul Afti Bakhtiar Afendy, David B. Cordes, Aidan P. McKay

**Affiliations:** a Centre of Chemical Synthesis & Polymer Technology Institute of Science Universiti Teknologi MARA Puncak Alam 42300 Puncak Alam Selangor Malaysia; b Faculty of Applied Sciences, Universiti Teknologi MARA, 40450 Shah Alam, Selangor, Malaysia; c EaStCHEM School of Chemistry, University of St Andrews, St Andrews, Fife KY16 9ST, United Kingdom; University of Aberdeen, United Kingdom

**Keywords:** crystal structure, oxopyrrolidine, racemic, hydrogen bond

## Abstract

In the title compound, C_17_H_22_N_2_O_5_ the pyrrolidine ring is almost planar and subtends a dihedral angle of 85.77 (7)° with the pendant phenyl ring. An intra­molecular N—H⋯O hydrogen bond generates an *S*(6) loop. In the crystal, the compound forms inversion dimers through O—H⋯O hydrogen bonds from the disordered hydroxyl group to either the hydroxyl or ester carbonyl O atom of the adjacent mol­ecule.

## Abstract 2

In the title compound, the pyrrolidine and phenyl rings are almost perpendicular and an intra­molecular N—H⋯O hydrogen bond occurs.

## Graphical abstract

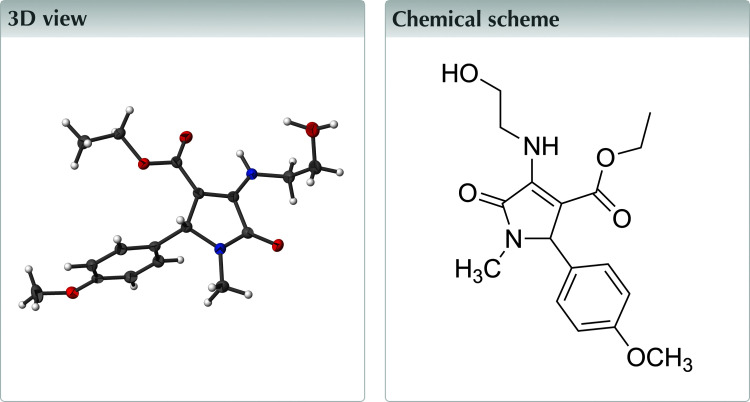

## Structure description

Mol­ecules bearing a γ-lactam moiety are receiving attention from researchers since examples of these compounds have been shown to exhibit potential medicinal uses, for example to inhibit the proteasome in cancer therapy (Ōmura & Crump, 2019 ▸), or to act as a potent inhibitor against methicillin-resistant *Staphylococcus aureus* (Miranda *et al.*, 2018 ▸; Wang *et al.*, 2020 ▸; Chen *et al.*, 2022 ▸). A facile method to prepare γ-lactams from readily available starting materials *via* one-pot multicomponent reactions has been reported in the literature (Metten *et al.*, 2006 ▸): these versatile precursors contain numerous functionalities that can be modified and transformed to other useful inter­mediates. In our previous work, a γ-lactam precursor was subjected to a Leuckart-type reaction (Rashid *et al.*, 2020 ▸) and herein we report the crystal structure of the title compound.

**Figure 1:**
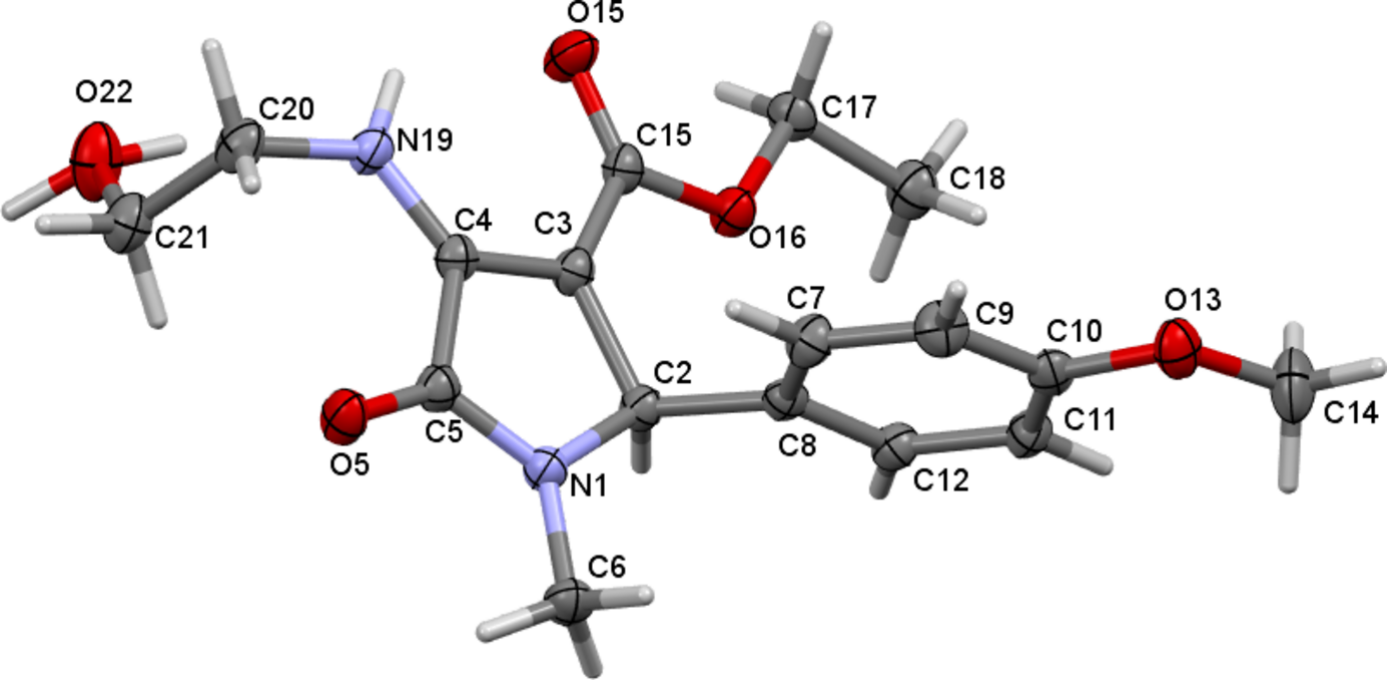
The mol­ecular structure of the title compound, showing displacement ellipsoids drawn at the 50% probability level. Both orientations of the disordered hydroxyl hydrogen atom are shown.

The title compound, C_17_H_22_N_2_O_5_, crystallizes in the monoclinic space group *P*2_1_/*n* with one mol­ecule in the asymmetric unit (Fig. 1 ▸). The five-membered pyrrolidine ring (C2–C5/N1) adopts a near planar conformation (r.m.s. deviation from planarity = 0.003 Å), with meth­oxy­benzene, ethyl ester and hy­droxy­ethyl amino substitutions at the 2, 3 and 4 ring positions, respectively. The dihedral angle between the pyrrolidine and phenyl rings is 85.77 (7)° and the N19—C20—C21—O22 torsion angle is −65.47 (16)°. The configuration of atom C2 in the asymmetric unit is *R* but crystal symmetry generates a racemic mixture. A weak intra­molecular N19—H19⋯O15 hydrogen bond (Table 1 ▸) occurs, which closes an *S*(6) ring. A similar feature was observed in the structure of ethyl 1-(2-hy­droxy­eth­yl)-4-[(4-meth­oxy­phen­yl)amino]-5-oxo-2,5-di­hydro-1*H*-pyrrole-3-carboxyl­ate (Abdul Rashid *et al.*, 2023 ▸).

**Figure 2:**
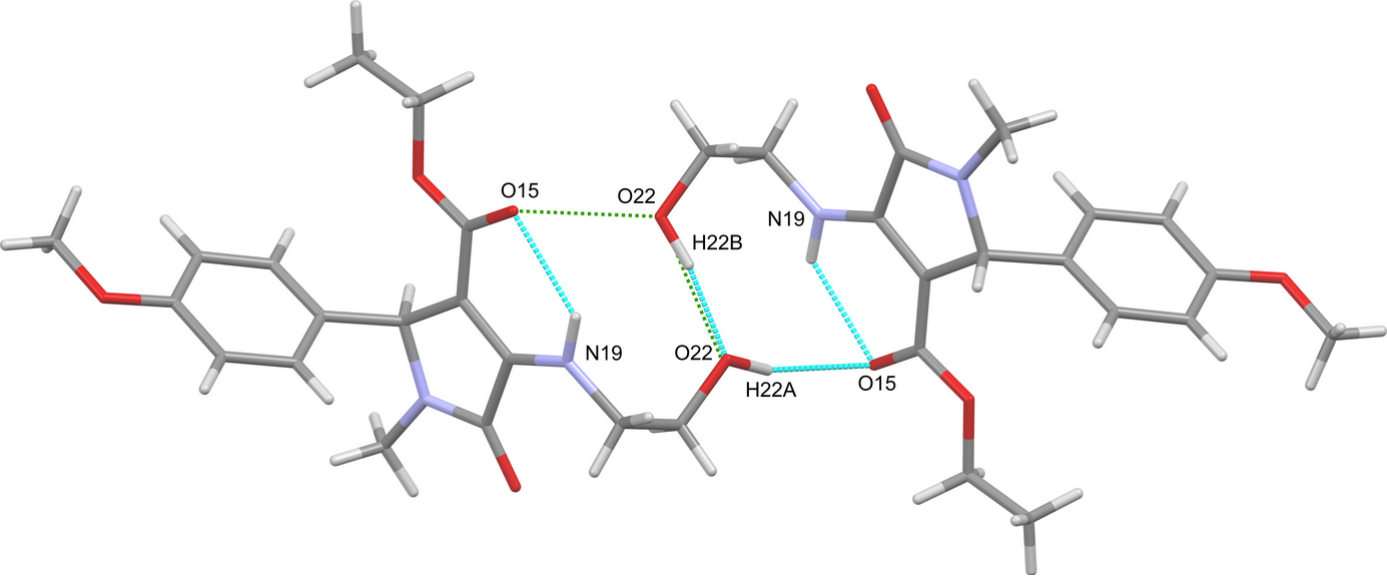
View of the supra­molecular dimers with both N—H⋯O intra­molecular and O—H⋯O inter­molecular hydrogen bonds. The disordered hydroxyl hydrogen atoms are shown in the ‘anti-clockwise’ conformation with the green dashed lines indicating the alternate ‘clockwise’ hydrogen-bonding scheme.

The terminal hydroxyl group of the hy­droxy­ethyl amino moiety exhibits positional disorder of its hydrogen atom. Both positions correspond to inter­molecular O—H⋯O hydrogen bonds to either the hydroxyl (O22) or ester carbonyl (O15) oxygen atom, of a neighbouring mol­ecule thereby forming 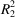
(11) rings that are either ‘anti-clockwise’ or ‘clockwise’ (Fig. 2 ▸). These dimers pack into the overall structure through a variety of weak C—H⋯O non-classical hydrogen bonds (Table 1 ▸).

## Synthesis and crystallization

The γ-lactam precursor, ethyl 4-hy­droxy-2-(4-meth­oxy­phen­yl)-1-methyl-5-oxo-2,5-di­hydro-1*H*-pyrrole-3-carboxyl­ate was synthesized following the reported method for related compounds (Rashid *et al.*, 2020 ▸). The title compound was prepared by adding ethano­lamine (0.25 ml, 4.12 mmol) to a solution of the γ-lactam precursor (1.00 g, 3.43 mmol) and formic acid (0.21 ml, 5.49 mmol) in ethanol (25 ml) and allowed to reflux for 12 h. After completion of the reaction, the solution was removed *in vacuo* and the crude product was dissolved in ethyl acetate, which was washed with water. The combined organic layers were dried over anhydrous MgSO_4_ before being concentrated under reduced pressure to yield a solid precipitate. Further washing of the precipitate with diethyl ether furnished the title compound as a dark-yellow solid (yield: 0.69 g, 60%). m.p. 89–90°C; IR (KBr, ν, cm^−1^): 3478 (NH), 1692 (C=O), 1621 (C=C), 1242 (C—N); ^1^H NMR (400 MHz, CDCl_3_ -*d_1_*) δ 7.03 (*d*, *J* = 8.7 Hz, 2H, CHAr), 6.80 (*d*, *J* = 8.7 Hz, 2H, CHAr), 4.87 (*s*, 1H, ArC*H*NCH_3_), 4.10–3.90 (*m*, 4H, OCH_2_ & C*H_2_*OH), 3.76–3.74 (*m*, 5H, OCH_3_ & NHC*H_2_*), 2.70 (*s*, 3H, NCH_3_), 1.01 (*t*, *J* = 7.1 Hz, 3H, CH_3_); ^13^C NMR (100 MHz, CDCl_3_ -*d_1_*) δ 165.9 (C=O), 165.5 (C=O), 159.4 (quat. Ar*C*), 147.6 (C—N), 129.0 (CHAr), 128.8 (quat. Ar*C*), 113.8 (CHAr), 103.6 (*C*CO), 63.4 (*C*H_2_OH), 63.2 (OCH_3_), 59.5 (OCH_2_), 55.3 (Ar*C*HNCH_3_), 44.6 (NH*C*H_2_), 27.6 (NCH_3_), 14.1 (CH_3_); CHN: found C, 59.64; H, 6.54; N, 7.74 requires C, 61.07; H, 6.63; N, 8.38%; LCMS (ESI): calculated for C_17_H_22_N_2_O_5_ 357.1 [*M* + Na]^+^, found 357.1. Crystals suitable for X-ray diffraction were grown by slow evaporation of an ethyl acetate solution at room temperature.

## Refinement

Crystal data, data collection and structure refinement details are summarized in Table 2 ▸. The N– and O-bound hydrogen atoms were located in a difference map and refined isotropically with distance restraints. The OH hydrogen atom was found to be disordered over two positions, its occupancy was fixed at 1/2, with *U*_eq_ riding on the parent atom.

## Supplementary Material

Crystal structure: contains datablock(s) I. DOI: 10.1107/S2414314624012227/hb4500sup1.cif

Structure factors: contains datablock(s) I. DOI: 10.1107/S2414314624012227/hb4500Isup2.hkl

Supporting information file. DOI: 10.1107/S2414314624012227/hb4500Isup3.cml

CCDC reference: 2411088

Additional supporting information:  crystallographic information; 3D view; checkCIF report

## Figures and Tables

**Table 1:** Hydrogen-bond geometry (Å, °)

*D*—H⋯*A*	*D*—H	H⋯*A*	*D*⋯*A*	*D*—H⋯*A*
N19—H19⋯O15	0.92 (1)	2.19 (2)	2.8595 (15)	129 (2)
O22—H22*A*⋯O15^i^	0.98 (1)	2.08 (2)	3.0131 (16)	157 (4)
O22—H22*B*⋯O22^i^	0.98 (2)	1.83 (2)	2.796 (2)	167 (4)
C6—H6*B*⋯O22^ii^	0.98	2.57	3.4152 (18)	144
C8—H8⋯O13^iii^	0.95	2.34	3.2556 (17)	162

Symmetry codes: (i) 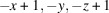; (ii) 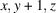; (iii) 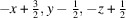.

**Table 2:** Experimental details

Crystal data	
Chemical formula	C_17_H_22_N_2_O_5_
*M*_r_	334.36
Crystal system, space group	Monoclinic, *P*2_1_/*n*
Temperature (K)	100
*a*, *b*, *c* (Å)	10.17216 (8), 9.24320 (6), 17.64603 (14)
β (°)	101.5111 (8)
*V* (Å^3^)	1625.77 (2)
*Z*	4
Radiation type	Cu *K*α
μ (mm^−1^)	0.84
Crystal size (mm)	0.09 × 0.07 × 0.01

Data collection	
Diffractometer	Rigaku XtaLAB P200K
Absorption correction	Multi-scan (*CrysAlis PRO*; Rigaku OD, 2024 ▸)
*T*_min_, *T*_max_	0.735, 1.000
No. of measured, independent and observed [*I* > 2σ(*I*)] reflections	57922, 3336, 3074
*R*_int_	0.071
(sin θ/λ)_max_ (Å^−1^)	0.628

Refinement	
*R*[*F*^2^ > 2σ(*F*^2^)], *wR*(*F*^2^), *S*	0.040, 0.106, 1.06
No. of reflections	3336
No. of parameters	230
No. of restraints	3
H-atom treatment	H atoms treated by a mixture of independent and constrained refinement
Δρ_max_, Δρ_min_ (e Å^−3^)	0.28, −0.27

Computer programs: *CrysAlis PRO* (Rigaku OD, 2024 ▸), *SHELXT2018/2* (Sheldrick, 2015*a* ▸), *SHELXL2019/3* (Sheldrick, 2015*b* ▸), *Mercury* (Macrae *et al.*, 2020 ▸), *OLEX2* (Dolomanov *et al.*, 2009 ▸) and *publCIF* (Westrip, 2010 ▸).

## full crystallographic data

### Ethyl 4-[(2-hydroxyethyl)amino]-2-(4-methoxyphenyl)-1-methyl-5-oxo-2,5-dihydro-1H-pyrrole-3-carboxylate Crystal data

**Table:**

C_17_H_22_N_2_O_5_	*F*(000) = 712
*M**_r_* = 334.36	*D*_x_ = 1.366 Mg m^−^^3^
Monoclinic, *P*2_1_/*n*	Cu *K*α radiation, λ = 1.54184 Å
*a* = 10.17216 (8) Å	Cell parameters from 26738 reflections
*b* = 9.24320 (6) Å	θ = 4.6–75.3°
*c* = 17.64603 (14) Å	µ = 0.84 mm^−^^1^
β = 101.5111 (8)°	*T* = 100 K
*V* = 1625.77 (2) Å^3^	Plate, colourless
*Z* = 4	0.09 × 0.07 × 0.01 mm

### Ethyl 4-[(2-hydroxyethyl)amino]-2-(4-methoxyphenyl)-1-methyl-5-oxo-2,5-dihydro-1H-pyrrole-3-carboxylate Data collection

**Table:**

Rigaku XtaLAB P200K diffractometer	3336 independent reflections
Radiation source: Rotating Anode, Rigaku MM-007HF	3074 reflections with *I* > 2σ(*I*)
Rigaku Osmic Confocal Optical System monochromator	*R*_int_ = 0.071
Detector resolution: 5.8140 pixels mm^-1^	θ_max_ = 75.6°, θ_min_ = 4.7°
shutterless scans	*h* = −12→12
Absorption correction: multi-scan (CrysAlisPro; Rigaku OD, 2024)	*k* = −11→11
*T*_min_ = 0.735, *T*_max_ = 1.000	*l* = −22→22
57922 measured reflections	

### Ethyl 4-[(2-hydroxyethyl)amino]-2-(4-methoxyphenyl)-1-methyl-5-oxo-2,5-dihydro-1H-pyrrole-3-carboxylate Refinement

**Table:**

Refinement on *F*^2^	Primary atom site location: dual
Least-squares matrix: full	Hydrogen site location: mixed
*R*[*F*^2^ > 2σ(*F*^2^)] = 0.040	H atoms treated by a mixture of independent and constrained refinement
*wR*(*F*^2^) = 0.106	*w* = 1/[σ^2^(*F*_o_^2^) + (0.0505*P*)^2^ + 0.7868*P*] where *P* = (*F*_o_^2^ + 2*F*_c_^2^)/3
*S* = 1.06	(Δ/σ)_max_ < 0.001
3336 reflections	Δρ_max_ = 0.28 e Å^−^^3^
230 parameters	Δρ_min_ = −0.27 e Å^−^^3^
3 restraints	

### Ethyl 4-[(2-hydroxyethyl)amino]-2-(4-methoxyphenyl)-1-methyl-5-oxo-2,5-dihydro-1H-pyrrole-3-carboxylate Special details

**Table:**

Geometry. All esds (except the esd in the dihedral angle between two l.s. planes) are estimated using the full covariance matrix. The cell esds are taken into account individually in the estimation of esds in distances, angles and torsion angles; correlations between esds in cell parameters are only used when they are defined by crystal symmetry. An approximate (isotropic) treatment of cell esds is used for estimating esds involving l.s. planes.
Refinement. Hydrogen atoms were placed in calculated positions except the on hydroxyl and amine groups (N19 and O22) which were located from F_map_ and refined subject to distance restraints and the U_eq_ of the hydroxyl hydrogens riding on O22. Hydrogens on O22 were observed in two distinct hydrogen bonding locations, both of which are modelled with occupancy fixed at 0.5 and H22B in part -1 as it was orientated towards a symmetry related H22B—O22.

### Ethyl 4-[(2-hydroxyethyl)amino]-2-(4-methoxyphenyl)-1-methyl-5-oxo-2,5-dihydro-1H-pyrrole-3-carboxylate Fractional atomic coordinates and isotropic or equivalent isotropic displacement parameters (Å^2^)

**Table:**

	*x*	*y*	*z*	*U*_iso_*/*U*_eq_	Occ. (<1)
O5	0.34109 (10)	0.32829 (11)	0.29196 (6)	0.0283 (2)	
O13	0.93989 (10)	0.92848 (11)	0.31984 (6)	0.0289 (2)	
O15	0.80294 (10)	0.22664 (11)	0.49182 (6)	0.0316 (3)	
O16	0.80905 (9)	0.46054 (10)	0.52944 (5)	0.0236 (2)	
O22	0.40444 (12)	−0.06392 (13)	0.44164 (6)	0.0394 (3)	
H22A	0.321 (2)	−0.096 (5)	0.457 (2)	0.059*	0.5
H22B	0.480 (4)	−0.017 (5)	0.477 (2)	0.059*	0.5
N1	0.45573 (11)	0.51525 (12)	0.36200 (7)	0.0223 (2)	
N19	0.57207 (12)	0.15014 (12)	0.37782 (7)	0.0229 (3)	
H19	0.6474 (15)	0.1133 (19)	0.4098 (10)	0.035 (5)*	
C2	0.57805 (13)	0.54035 (14)	0.42004 (7)	0.0201 (3)	
H2	0.553384	0.575074	0.469032	0.024*	
C3	0.63422 (13)	0.38870 (14)	0.43190 (7)	0.0203 (3)	
C4	0.55360 (13)	0.29314 (14)	0.38504 (7)	0.0202 (3)	
C5	0.43611 (13)	0.37609 (15)	0.33929 (8)	0.0218 (3)	
C6	0.36725 (14)	0.63225 (15)	0.32925 (9)	0.0273 (3)	
H6A	0.410681	0.690047	0.294676	0.041*	
H6B	0.348131	0.693750	0.370983	0.041*	
H6C	0.283186	0.591964	0.299979	0.041*	
C7	0.67047 (13)	0.64984 (14)	0.39345 (7)	0.0200 (3)	
C8	0.70629 (14)	0.63505 (15)	0.32152 (8)	0.0247 (3)	
H8	0.670204	0.557712	0.288350	0.030*	
C9	0.79374 (14)	0.73181 (16)	0.29807 (8)	0.0266 (3)	
H9	0.816202	0.721865	0.248590	0.032*	
C10	0.84893 (13)	0.84382 (14)	0.34690 (8)	0.0226 (3)	
C11	0.81191 (14)	0.86208 (14)	0.41785 (8)	0.0235 (3)	
H11	0.847341	0.940002	0.450770	0.028*	
C12	0.72214 (13)	0.76474 (14)	0.44028 (8)	0.0221 (3)	
H12	0.695973	0.777571	0.488603	0.027*	
C14	1.00316 (18)	1.03976 (19)	0.36932 (11)	0.0411 (4)	
H14A	1.062776	1.095104	0.342862	0.062*	
H14B	1.055617	0.996804	0.416585	0.062*	
H14C	0.934820	1.104289	0.382711	0.062*	
C15	0.75500 (13)	0.34857 (14)	0.48560 (7)	0.0212 (3)	
C17	0.93178 (14)	0.43252 (15)	0.58571 (8)	0.0245 (3)	
H17A	1.003709	0.398723	0.559586	0.029*	
H17B	0.916399	0.357827	0.623200	0.029*	
C18	0.96974 (15)	0.57457 (16)	0.62607 (8)	0.0301 (3)	
H18A	0.982430	0.647720	0.587974	0.045*	
H18B	1.053341	0.562775	0.664254	0.045*	
H18C	0.898122	0.605610	0.652238	0.045*	
C20	0.47715 (15)	0.04845 (15)	0.33305 (8)	0.0267 (3)	
H20A	0.525209	−0.041922	0.325556	0.032*	
H20B	0.441995	0.090415	0.281357	0.032*	
C21	0.36072 (16)	0.01227 (17)	0.37099 (8)	0.0304 (3)	
H21A	0.315223	0.102708	0.381347	0.036*	
H21B	0.295103	−0.047698	0.335390	0.036*	

### Ethyl 4-[(2-hydroxyethyl)amino]-2-(4-methoxyphenyl)-1-methyl-5-oxo-2,5-dihydro-1H-pyrrole-3-carboxylate Atomic displacement parameters (Å^2^)

**Table:**

	*U*^11^	*U*^22^	*U*^33^	*U*^12^	*U*^13^	*U*^23^
O5	0.0270 (5)	0.0245 (5)	0.0287 (5)	−0.0031 (4)	−0.0058 (4)	0.0000 (4)
O13	0.0292 (5)	0.0295 (5)	0.0276 (5)	−0.0053 (4)	0.0047 (4)	0.0077 (4)
O15	0.0321 (6)	0.0207 (5)	0.0361 (6)	0.0034 (4)	−0.0076 (4)	−0.0020 (4)
O16	0.0247 (5)	0.0208 (5)	0.0222 (5)	−0.0009 (4)	−0.0028 (4)	0.0002 (4)
O22	0.0470 (7)	0.0409 (6)	0.0277 (6)	−0.0162 (5)	0.0015 (5)	0.0051 (5)
N1	0.0201 (5)	0.0198 (6)	0.0251 (6)	0.0000 (4)	0.0000 (4)	0.0006 (4)
N19	0.0243 (6)	0.0181 (5)	0.0240 (6)	−0.0011 (4)	−0.0007 (5)	0.0002 (4)
C2	0.0211 (6)	0.0192 (6)	0.0189 (6)	−0.0005 (5)	0.0015 (5)	−0.0004 (5)
C3	0.0228 (6)	0.0191 (6)	0.0189 (6)	−0.0016 (5)	0.0037 (5)	0.0007 (5)
C4	0.0219 (6)	0.0207 (6)	0.0180 (6)	−0.0015 (5)	0.0043 (5)	0.0019 (5)
C5	0.0224 (6)	0.0205 (6)	0.0220 (6)	−0.0010 (5)	0.0034 (5)	0.0019 (5)
C6	0.0244 (7)	0.0223 (7)	0.0327 (7)	0.0031 (5)	0.0000 (6)	0.0026 (6)
C7	0.0202 (6)	0.0184 (6)	0.0198 (6)	0.0018 (5)	0.0004 (5)	0.0018 (5)
C8	0.0293 (7)	0.0234 (7)	0.0202 (6)	−0.0025 (6)	0.0020 (5)	−0.0028 (5)
C9	0.0305 (7)	0.0307 (7)	0.0191 (6)	−0.0009 (6)	0.0059 (5)	0.0020 (5)
C10	0.0217 (6)	0.0208 (6)	0.0237 (6)	0.0010 (5)	0.0008 (5)	0.0075 (5)
C11	0.0259 (7)	0.0188 (6)	0.0237 (6)	−0.0016 (5)	0.0000 (5)	−0.0013 (5)
C12	0.0247 (7)	0.0211 (6)	0.0200 (6)	0.0006 (5)	0.0033 (5)	−0.0006 (5)
C14	0.0409 (9)	0.0382 (9)	0.0460 (9)	−0.0194 (7)	0.0129 (7)	−0.0030 (7)
C15	0.0235 (6)	0.0199 (6)	0.0203 (6)	−0.0035 (5)	0.0042 (5)	0.0012 (5)
C17	0.0222 (7)	0.0261 (7)	0.0224 (6)	−0.0003 (5)	−0.0020 (5)	0.0021 (5)
C18	0.0309 (8)	0.0290 (8)	0.0268 (7)	−0.0033 (6)	−0.0026 (6)	−0.0018 (6)
C20	0.0316 (7)	0.0222 (7)	0.0239 (7)	−0.0022 (6)	−0.0004 (6)	−0.0030 (5)
C21	0.0356 (8)	0.0259 (7)	0.0285 (7)	−0.0056 (6)	0.0037 (6)	−0.0017 (6)

### Ethyl 4-[(2-hydroxyethyl)amino]-2-(4-methoxyphenyl)-1-methyl-5-oxo-2,5-dihydro-1H-pyrrole-3-carboxylate Geometric parameters (Å, º)

**Table:**

O5—C5	1.2267 (16)	C7—C8	1.3955 (19)
O13—C10	1.3684 (16)	C7—C12	1.3833 (18)
O13—C14	1.4178 (19)	C8—H8	0.9500
O15—C15	1.2242 (17)	C8—C9	1.382 (2)
O16—C15	1.3424 (16)	C9—H9	0.9500
O16—C17	1.4549 (15)	C9—C10	1.392 (2)
O22—H22A	0.983 (5)	C10—C11	1.3879 (19)
O22—H22B	0.983 (19)	C11—H11	0.9500
O22—C21	1.4236 (18)	C11—C12	1.3944 (19)
N1—C2	1.4636 (16)	C12—H12	0.9500
N1—C5	1.3502 (17)	C14—H14A	0.9800
N1—C6	1.4512 (17)	C14—H14B	0.9800
N19—H19	0.922 (14)	C14—H14C	0.9800
N19—C4	1.3447 (17)	C17—H17A	0.9900
N19—C20	1.4613 (17)	C17—H17B	0.9900
C2—H2	1.0000	C17—C18	1.507 (2)
C2—C3	1.5124 (18)	C18—H18A	0.9800
C2—C7	1.5180 (18)	C18—H18B	0.9800
C3—C4	1.3659 (18)	C18—H18C	0.9800
C3—C15	1.4423 (18)	C20—H20A	0.9900
C4—C5	1.5109 (18)	C20—H20B	0.9900
C6—H6A	0.9800	C20—C21	1.510 (2)
C6—H6B	0.9800	C21—H21A	0.9900
C6—H6C	0.9800	C21—H21B	0.9900
			
C10—O13—C14	117.09 (11)	O13—C10—C11	124.70 (12)
C15—O16—C17	116.94 (10)	C11—C10—C9	120.04 (12)
H22A—O22—H22B	125 (4)	C10—C11—H11	120.4
C21—O22—H22A	105 (3)	C10—C11—C12	119.25 (12)
C21—O22—H22B	114 (3)	C12—C11—H11	120.4
C5—N1—C2	114.42 (11)	C7—C12—C11	121.20 (12)
C5—N1—C6	123.27 (11)	C7—C12—H12	119.4
C6—N1—C2	122.25 (11)	C11—C12—H12	119.4
C4—N19—H19	114.8 (12)	O13—C14—H14A	109.5
C4—N19—C20	126.42 (12)	O13—C14—H14B	109.5
C20—N19—H19	118.2 (12)	O13—C14—H14C	109.5
N1—C2—H2	109.3	H14A—C14—H14B	109.5
N1—C2—C3	101.24 (10)	H14A—C14—H14C	109.5
N1—C2—C7	112.45 (10)	H14B—C14—H14C	109.5
C3—C2—H2	109.3	O15—C15—O16	123.23 (12)
C3—C2—C7	114.87 (11)	O15—C15—C3	124.58 (12)
C7—C2—H2	109.3	O16—C15—C3	112.19 (11)
C4—C3—C2	110.54 (11)	O16—C17—H17A	110.6
C4—C3—C15	124.14 (12)	O16—C17—H17B	110.6
C15—C3—C2	125.31 (11)	O16—C17—C18	105.76 (11)
N19—C4—C3	127.92 (13)	H17A—C17—H17B	108.7
N19—C4—C5	123.97 (12)	C18—C17—H17A	110.6
C3—C4—C5	108.10 (11)	C18—C17—H17B	110.6
O5—C5—N1	126.51 (13)	C17—C18—H18A	109.5
O5—C5—C4	127.79 (12)	C17—C18—H18B	109.5
N1—C5—C4	105.69 (11)	C17—C18—H18C	109.5
N1—C6—H6A	109.5	H18A—C18—H18B	109.5
N1—C6—H6B	109.5	H18A—C18—H18C	109.5
N1—C6—H6C	109.5	H18B—C18—H18C	109.5
H6A—C6—H6B	109.5	N19—C20—H20A	108.9
H6A—C6—H6C	109.5	N19—C20—H20B	108.9
H6B—C6—H6C	109.5	N19—C20—C21	113.26 (12)
C8—C7—C2	120.40 (12)	H20A—C20—H20B	107.7
C12—C7—C2	120.81 (11)	C21—C20—H20A	108.9
C12—C7—C8	118.79 (12)	C21—C20—H20B	108.9
C7—C8—H8	119.7	O22—C21—C20	111.24 (13)
C9—C8—C7	120.63 (13)	O22—C21—H21A	109.4
C9—C8—H8	119.7	O22—C21—H21B	109.4
C8—C9—H9	120.0	C20—C21—H21A	109.4
C8—C9—C10	120.03 (12)	C20—C21—H21B	109.4
C10—C9—H9	120.0	H21A—C21—H21B	108.0
O13—C10—C9	115.26 (12)		
			
O13—C10—C11—C12	−177.32 (12)	C5—N1—C2—C7	122.29 (12)
N1—C2—C3—C4	0.97 (14)	C6—N1—C2—C3	−177.89 (12)
N1—C2—C3—C15	−178.34 (12)	C6—N1—C2—C7	−54.80 (16)
N1—C2—C7—C8	−50.65 (16)	C6—N1—C5—O5	−3.4 (2)
N1—C2—C7—C12	130.23 (13)	C6—N1—C5—C4	177.42 (12)
N19—C4—C5—O5	2.1 (2)	C7—C2—C3—C4	−120.45 (12)
N19—C4—C5—N1	−178.77 (12)	C7—C2—C3—C15	60.24 (17)
N19—C20—C21—O22	−65.47 (16)	C7—C8—C9—C10	1.2 (2)
C2—N1—C5—O5	179.51 (13)	C8—C7—C12—C11	−2.1 (2)
C2—N1—C5—C4	0.36 (15)	C8—C9—C10—O13	176.51 (12)
C2—C3—C4—N19	178.21 (13)	C8—C9—C10—C11	−2.7 (2)
C2—C3—C4—C5	−0.81 (14)	C9—C10—C11—C12	1.8 (2)
C2—C3—C15—O15	−176.13 (13)	C10—C11—C12—C7	0.6 (2)
C2—C3—C15—O16	4.74 (18)	C12—C7—C8—C9	1.2 (2)
C2—C7—C8—C9	−177.94 (12)	C14—O13—C10—C9	−177.18 (14)
C2—C7—C12—C11	177.04 (12)	C14—O13—C10—C11	2.0 (2)
C3—C2—C7—C8	64.43 (16)	C15—O16—C17—C18	179.91 (11)
C3—C2—C7—C12	−114.69 (14)	C15—C3—C4—N19	−2.5 (2)
C3—C4—C5—O5	−178.84 (13)	C15—C3—C4—C5	178.51 (12)
C3—C4—C5—N1	0.30 (14)	C17—O16—C15—O15	1.01 (19)
C4—N19—C20—C21	−74.66 (17)	C17—O16—C15—C3	−179.84 (10)
C4—C3—C15—O15	4.7 (2)	C20—N19—C4—C3	173.28 (13)
C4—C3—C15—O16	−174.48 (12)	C20—N19—C4—C5	−7.8 (2)
C5—N1—C2—C3	−0.80 (14)		

### Ethyl 4-[(2-hydroxyethyl)amino]-2-(4-methoxyphenyl)-1-methyl-5-oxo-2,5-dihydro-1H-pyrrole-3-carboxylate Hydrogen-bond geometry (Å, º)

**Table:**

*D*—H···*A*	*D*—H	H···*A*	*D*···*A*	*D*—H···*A*
N19—H19···O15	0.92 (1)	2.19 (2)	2.8595 (15)	129 (2)
O22—H22*A*···O15^i^	0.98 (1)	2.08 (2)	3.0131 (16)	157 (4)
O22—H22*B*···O22^i^	0.98 (2)	1.83 (2)	2.796 (2)	167 (4)
C6—H6*B*···O22^ii^	0.98	2.57	3.4152 (18)	144
C8—H8···O13^iii^	0.95	2.34	3.2556 (17)	162

Symmetry codes: (i) −*x*+1, −*y*, −*z*+1; (ii) *x*, *y*+1, *z*; (iii) −*x*+3/2, *y*−1/2, −*z*+1/2.
